# The Independent and Joint Associations of Whole Grain and Refined Grain with Total Mortality among Breast Cancer Survivors: A Prospective Cohort Study

**DOI:** 10.3390/nu14163333

**Published:** 2022-08-15

**Authors:** Deepali Karina Ernest, Hector Lemus, Fang-Chi Hsu, John P. Pierce, Tianying Wu

**Affiliations:** 1 Division of Epidemiology and Biostatistics, School of Public Health, San Diego State University, San Diego, CA 92182, USA; 2 Department of Biostatistics and Data Science, Division of Public Health Sciences, Wake Forest University School of Medicine, Winston-Salem, NC 27101, USA; 3 Herbert Wertheim School of Public Health and Human Longevity Science, University of California, San Diego, CA 92093, USA; 4 Moores Cancer Center, School of Medicine, University of California, San Diego, CA 92037, USA

**Keywords:** whole grains, refined grains, mortality, breast cancer survivors, digestive system

## Abstract

Breast cancer survivors often have a reduced digestive capacity to digest whole grains due to cancer treatment. The purpose of this study was to investigate the independent and joint associations of whole grain and refined grain consumption with total mortality among breast cancer survivors. We studied a cohort of 3081 female breast cancer survivors who provided demographic, dietary, and lifestyle data at baseline, year 1 and year 4. Mortality was assessed via semi-annual telephone interviews and confirmed by the National Death Index (NDI) and death certificates. We assessed the associations of whole grain and refined grain with incident of mortalities using Cox proportional hazards models. Increased whole grain consumption was marginally associated with an increased risk of total mortality (*p* = 0.07) but was not significantly associated with breast cancer-specific mortality (*p* = 0.55). An increased intake of refined grains was associated with an increased risk of both total (HR = 1.74; 95% CI,1.17 to 2.59) and breast cancer-specific mortality (HR = 1.16; 95% CI, 1.08 to 1.26). Furthermore, we examined the joint associations of whole grain and refined grain with total mortality. Among those with a high consumption of refined grain, those with high consumption of whole grain had a higher risk of total mortality (HR = 1.52, 95% CI, 1.07 to 2.14) than those with a low consumption of whole grain. Increased consumption of whole grains may exacerbate the adverse associations of refined grain with mortality among breast cancer survivors. Our findings indicate the need to revisit current dietary guidelines for breast cancer survivors regarding whole grain intake.

## 1. Introduction

Breast cancer is the most prevalent form of cancer and one of the leading causes of cancer-related mortality among women in the United States (US) [1,2,3]. Approximately 287,850 cases of invasive breast cancer and 51,400 cases of non-invasive breast cancer are predicted to be diagnosed in the U.S in 2022 [4]. Recent advancements in cancer therapies have led to a steady decline in breast cancer mortality; however, these therapies have adverse effects. Chemotherapy weakens the digestive system, causing abdominal pain, bloating, and nausea; radiation therapy triggers cell necrosis, apoptosis, and persistent activation of cytokines in the submucosa leading to acute damages to the gut [5,6]. Therefore, specialized dietary guidelines for breast cancer survivors are needed to better suit their gastrointestinal abilities and nutrition requirements [1].

Current dietary guidelines for cancer survivors recommend consuming more than 5 servings of fruits and cruciferous vegetables (including garlic) and 3–6 servings of whole grains per day, ample protein (nuts, seeds, legumes, soy, and poultry), and limited intake of alcohol, refined sugars, fats, and red or cured meat [7,8]. However, these guidelines may have been generated from the general population rather than breast cancer survivors [7]. Furthermore, results based on the general population examining the associations of whole grain intake and risk of risk of breast cancer are not consistent [9].

Precision nutrition emphasizes “one size does not fit all”, which is one of the premier priorities in the 2020 to 2030 NIH Nutrition Strategic plan [10]. Hence, studies focusing on breast cancer survivors are needed to generate guidelines tailored to them. 

The “3–6 servings of whole grains per day” recommendation in the current dietary guidelines may not be suitable for breast cancer survivors because previous studies determining the associations of whole grains and refined grains with prognoses were mainly generated from non-cancer survivors [7,8]. Furthermore, whether whole grains and refined grains have independent and joint associations with mortality has never been studied. Considering the damages caused by cancer treatment, rigorous prospective studies are needed. 

Our study aims to address these gaps in the literature by leveraging the Women’s Healthy Eating and Living (WHEL) study, which collected detailed dietary and lifestyle information and mortality data longitudinally. We investigated the independent and joint associations of whole grains and refined grains with the risk of mortality among female breast cancer survivors in the US. 

## 2. Materials and Methods

### 2.1. Study Design and Population

The WHEL study was a multi-center US randomized control trial conducted to determine whether a diet rich in vegetables, fruits, and fiber and low in dietary fat reduced the risk of recurrence and new primary breast cancer and total mortality among women previously treated for early-stage breast cancer. Between 1995 to 2000, 3088 participants between the ages of 18 to 70 were enrolled using tumor registries, local oncologists, community outreach events, and mass media advertisement, and followed through to 2006. Detailed inclusion and exclusion criteria were described in the original WHEL study [11]. Briefly, participants had to be diagnosed with stage I, II, or IIIA cancer, not actively receiving treatment and not experiencing a recurrence or new cancer. Participants were excluded if they presented with cirrhosis, pregnancy, life-threatening diseases, medical conditions (other than breast cancer), comorbidities requiring medication, or a diet identified as a contraindication to a high fiber diet or receiving estrogen replacement therapy [12]. We treated this study as an observational cohort study and excluded women who had missing whole or refined grain data at baseline (*n* = 7), resulting in a total of 3081 survivors.

### 2.2. Assessment of Total Dietary Intakes, Whole Grain, and Refined Grain Intakes

Four prescheduled 24 h recalls were collected at each visit. Dietary intakes from each 24 h recall were calculated and the average intakes of the four 24 h recalls from each visit were used for final data analyses. Our study used the 24 h dietary data collected at baseline, year 1 and year 4. A multi-pass software-driven recall protocol, named the Nutritional Data System (NDS-R, 1994–2006, 91 University of Minnesota, Minneapolis, MN, USA), was used to analyze the foods and nutrients. The intake of whole grains was extracted from whole-grain-contributing foods such as cereal, brown rice, oatmeal, and whole-wheat bread. Similarly, refined grain was estimated from refined-grain contributing foods such as white bread, enriched grains, and white-flour-based food. These final estimates were quantified as servings/day.

### 2.3. Assessment of Mortality 

Total mortality was initially assessed through semi-annual telephone interviews by clinical site coordinators or a designee. All mortality information was confirmed by the National Death Index (NDI) using Social Security number, name, and date of birth. Mortalities among participants lost to follow-up were also obtained using the NDI and death certificates. Follow-up time was censored at either the time of a participant’s death, the last date they were contacted, or the date they completed the study.

### 2.4. Assessment of Demographic Factors, Lifestyle Habits, and Treatment Regimens

Along with 24 h dietary recalls, study participants were mailed a set of questionnaires (regarding personal habits, quality of life, clinical measures, and health symptoms) prior to each clinic visit at baseline, year 1 and year 4. These questionnaires assessed multiple characteristics and demographics such as age at diagnosis (years), weight (kilograms), height (centimeters), waist circumference (centimeters), marital status (single, married, other), education level (high school or less, high school to college, post college), smoking status (never, former, current), menopausal status (premenopausal, perimenopausal, postmenopausal), employment status (unemployed, employed), physical activity, cancer stage (I, II, IIIA), type of cancer therapy received (radiation therapy, chemotherapy), hormone receptor status (e.g., estrogen receptor positive/progesterone receptor positive (ER+/PR+)), and symptoms of digestive disorders. 

### 2.5. Statistical Analysis

We used chi-square tests for categorical variables, t-tests/analysis of variance (ANOVA) for normally distributed continuous variables and Wilcoxon rank sum test or Kruskal–Wallis for non-normally distributed continuous variables to estimate unadjusted associations between general baseline characteristics and total mortality, and between baseline characteristics and quartiles of whole grain intake. The associations of refined grain and whole grain intakes with total and breast-cancer-specific mortality were assessed using Cox proportional hazards models. The proportional hazards assumption was tested prior to analyses using the Kolmogorov-type Supremum Test [13]. The Kolmogorov-type Supremum Test calculated the differences between the observed and expected residues based on simulations; a non-significant *p*-value indicates that impacts of covariates are constant over time, and the proportional hazards assumption is met [14]. Additionally, we utilized the counting technique to account for the time-varying measurements of whole and refined grain data [15]. 

For total mortality, follow-up times for participants who did not die were calculated from study entry to loss to follow-up or to the end of the follow-up period, whichever came first. For participants who died, follow-up time was calculated from the study entry to the time of death. For breast-cancer-specific mortality, follow-up time was calculated from the study entry to breast-cancer-specific death; death from other causes was treated as a competing risk. We adjusted for the following covariates: age at diagnosis, smoking status, cancer stage, chemotherapy status, radiation status, body mass index (BMI), physical activity, hormone receptor status, menopausal status, whole grain/refined grain, and total calorie and protein intakes; among them, smoking status, BMI, physical activity, and menopausal status were time-varying covariates. Education and marital status were removed from the model as they had no bearing on the estimates of the main associations of interest. We conducted a multivariable-adjusted spline analysis using the RCS_Reg macro in SAS to determine the shape of the relationship between whole/refined grain and total mortality [16]. 

We assessed the joint associations of whole grain and refined grain with total mortality using a similar Cox proportional hazards model. Using their respective medians as the cut-off, we classified whole grain and refined grain into high- and low-groups, resulting in a four-level categorical variable. Women with high intakes of whole grains but low intakes of refined grains were treated as the reference group. 

## 3. Results

### 3.1. Baseline Characteristics

The length of follow-up time ranged from 1 to 11.2 years, and a total of 314 deaths occurred during this time. The median time to death was 7.1 years. As shown in Table 1, the mean age at randomization was 52.8 years and mean age at diagnosis was 50.7 years. Compared to women with no incidents of death, women who died from all causes had higher BMI, lower physical activity, and lower education level. A greater proportion of those who died received chemotherapy, were ER+/PR+, and were single. The *p*-values for these comparisons were <0.05.

Table 2 displays the patterns of baseline characteristics associated with whole grains. With an increase of whole grain intake, we observed a downward trend of refined grain consumption, fewer never smokers and higher levels of physical activity; *p*-values for these comparisons were <0.05. 

### 3.2. Independent Associations of Whole Grain and Refined Grain with Mortality

As illustrated in Table 3, the association of whole grain with total mortality was marginally significant (*p* = 0.07) when we assessed the *p*-value for trends and the point estimates for each quartile versus the reference category. Whole grain consumption was not significantly associated with breast-cancer-specific mortality. We reported a statistically significant positive association between refined grain and total/breast-cancer-specific mortality. Compared to women with refined grain intake in quartile 1, women with refined grain intakes in quartile 4 had 1.74-times the risk of total mortality (95% CI: 1.17 to 2.59) and 1.16-times the risk of breast-cancer-specific mortality (95% CI:1.08 to 1.26); *p*-values for trends were <0.05 for both outcomes. 

**Figure 1:**
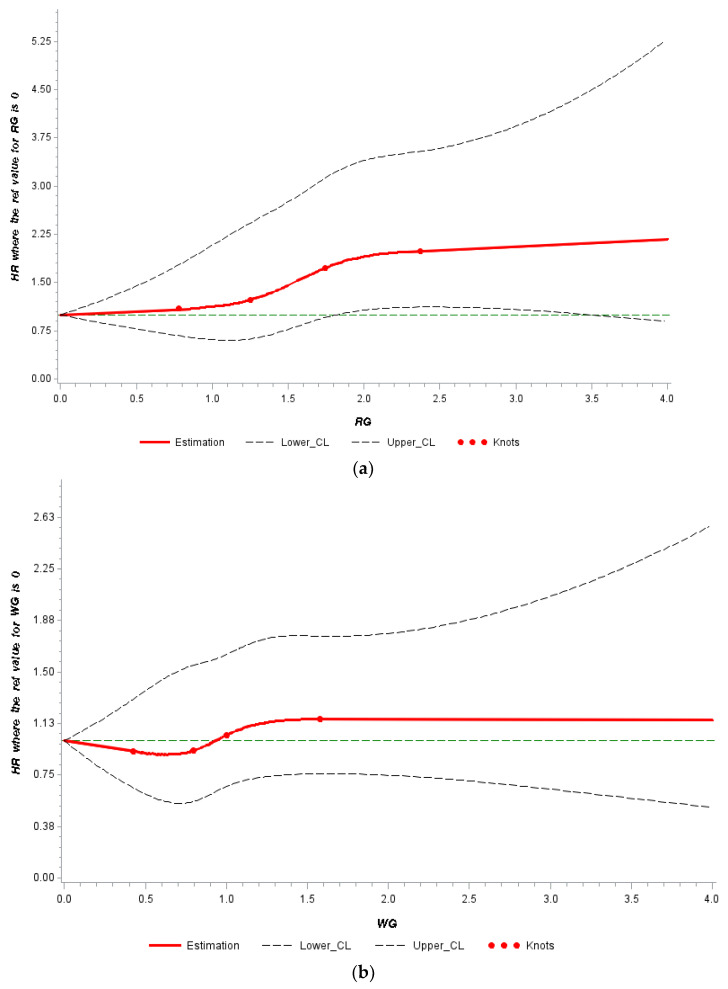
The spline models were adjusted for age at diagnosis, smoking status, cancer stage, chemotherapy, radiation therapy, total calorie and protein intakes, body mass index, menopausal status, hormone status and physical activity. Abbreviations: RG = refined grains, WG = whole grains. (**a**) The association between refined grain consumption and total mortality. (**b**) The association between whole grain consumption and total mortality.

The multivariable-adjusted spline analysis in Figure 1a illustrates the relationship between refined grain consumption and total mortality. It demonstrates a statistically significant association between refined grain intake and total mortality (*p* = 0.0027), and the test for non-linearity was insignificant (*p* = 0.17), which is in line with the results in Table 3. Figure 1b showed no association between whole grain and total mortality (*p* = 0.65), and the test for non-linearity was not significant (*p* = 0.76). We observed a slight, inverse relationship between whole grain and mortality when whole grain intakes were lower than 1 serving/day.

### 3.3. Joint Associations of Whole Grain and Refined Grain with Total Mortality

**Figure 2:**
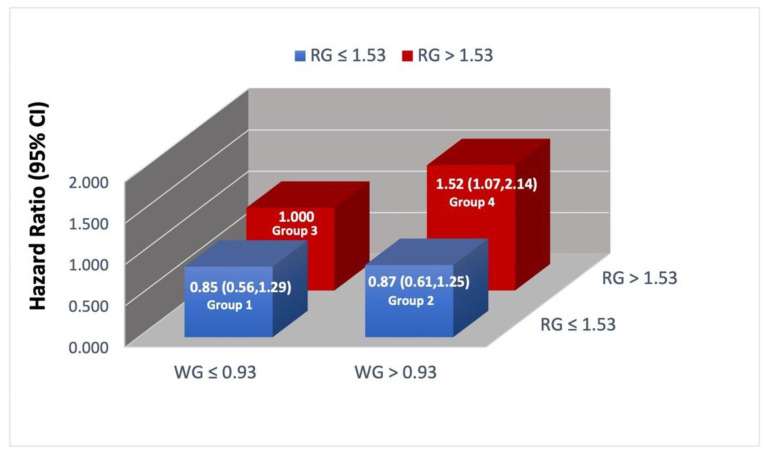
Joint associations of whole grains and refined grains with total mortality. The hazard ratios presented in this chart were adjusted for age at diagnosis, smoking status, menopause status, physical activity, body mass index, total calorie and protein intake, and cancer characteristics, namely chemotherapy, radiation therapy, cancer stage, and hormone status. Abbreviations: RG = refined grains; WG = whole grains.

As illustrated in Figure 2, when we treated women with high intakes of refined grain sand low intakes of whole grains as the reference group, women with the highest intake of both whole grains and refined grains had the largest risk of total mortality (HR = 1.52; 95% CI, 1.07 to 2.14); the hazard ratios for the other groups were not statistically different from the reference group. 

## 4. Discussion

Our study investigated the independent and joint associations of whole grains and refined grains with mortality among breast cancer survivors. We found a linear and positive relationship of refined grains with total mortality and breast-cancer-specific mortality. Whole grain consumption was marginally associated with total mortality but not with breast-cancer-specific mortality. The analyses of joint associations of whole grains and refined grains with total mortality highlighted the potential adverse effects of high intakes of whole grains on total mortality. Compared to those with a low intake of whole grains but high intake of refined grains, women with a high intake of both whole grains and refined grains had a hazard ratio of 1.52 (95% CI, 1.07 to 2.14). 

We found that increased whole grain intake was associated with increased risk of total mortality and exacerbated the adverse effects of refined grain on total mortality. Whole grain foods are rich sources of fiber, vitamin B, iron, folate, and other healthy bioactive components called phytochemicals [17,18], which may have antioxidant and radical scavenging properties. These bioactive substances can reduce one’s risk of chronic diseases such as type 2 diabetes, cardiovascular disease, and cancer [19,20,21]. Two large cohort studies, namely the Nurses’ Health Study (*n* = 74,341) and the Health Professionals Follow-Up Study (*n* = 43,744), found that increased whole grain intake was associated with a low risk of total and cardiovascular specific mortality among individuals free of cancer, stroke, and coronary heart disease (CHD) [19]. A meta-analysis of 20 prospective studies also documented that increased whole grain consumption was associated with a reduced risk of total mortality, cardiovascular-specific and cancer-specific mortality [22]. However, these studies focused on non-cancer survivors, who may have different digestive health conditions as compared to cancer survivors. Several cohort studies focusing on breast cancer survivors have documented that diets enriched with fiber, vegetables, and whole grains were associated with a reduced risk of total and cardiovascular specific mortality [23]; however, these studies assessed a dietary pattern rather than whole grain consumption alone and thus could not tease out the independent effect of whole grains on mortality.

While whole grain consumption provides health benefits such as reducing mortality and inflammation among generally healthy individuals [22,24,25], it can have adverse effects on individuals with impaired digestive systems. Several studies have found that common cancer treatments cause reduced gut integrity and impair gastrointestinal function among cancer patients. For instance, chemotherapy can induce acute dysbiosis [26]; radiation therapy generates reactive oxygen species resulting in persistent cytokine activation in the submucosa and acute damage in the gastrointestinal system [5]. Therefore, gastrointestinal damage caused by cancer treatments can make breast cancer patients more susceptible to the side effects of certain food groups such as whole grains. The following paragraph explores potential biological mechanisms discovered from current biomarker studies.

Biomarker-based research among breast cancer survivors has shown that impaired gut integrity is associated with whole grain consumption, severe gastrointestinal diseases, and breast cancer metastasis [27,28]. Claudin-2, a protein with physiologic properties of tight junctions, is a commonly used biomarker to gauge gut integrity. Increased claudin-2 has been observed in patients with sensitivity to gluten and wheat products; increased claudin-2 levels were observed in parallel with small intestinal epithelial defects and luminal leakage in these patients [29,30]. Claudin-2 is often upregulated in individuals suffering from gastrointestinal diseases such as Crohn’s disease, ulcerative colitis, and HIV-enteropathy. These diseases are caused by a leak flux mechanism due to intestinal barrier defects [31]. Furthermore, claudin-2 is detected in 52% of all breast carcinomas and is a known promoter of breast cancer metastasis [30,32,33]. It is thus important to account for the adverse effects of whole grain consumption on gastrointestinal systems among patients with impaired gut integrity such as breast cancer survivors.

We observed a positive and significant association between refined grains and total mortality/breast cancer-specific mortality. Refined grains are the most widely consumed grains in the US. They differ from whole grains in that they are highly processed, have a high glycemic index and glycemic load, and lack a fiber-rich bran and nutrient-filled germ. The absence of the phytochemical-dense germ degrades the grain’s nutritional ability to reduce oxidative stress and protect against cell damage [18,34]. A meta-analysis of observational studies found that high consumption of refined grains is associated with increased risks of type II diabetes, cardiovascular disease, and obesity among healthy individuals [35]. While many studies have investigated refined grains as part of a broader dietary pattern, very few have studied its independent effects on mortality. Only one study, The Prospective Urban and Rural Epidemiology Study (*n*= 148,858), documented a 27% increase in the risk of total mortality associated with consuming ≥ 7 servings/day of refined grains (as a distinct food group) among healthy individuals [36]. However, studies focusing on independent associations of refined grains with mortality among breast cancer survivors are limited. In the US, refined grains are often consumed with unhealthy diets consisting of highly processed foods, sugar-rich beverages, and large quantities of cured meat [37]; refined grains have been assessed along with these unhealthy foods as an unhealthy dietary pattern with total mortality among breast cancer survivors [38,39]. These findings raise questions as to whether consumption of refined grains with unhealthy diets overestimates the adverse effects of refined grains.

When we evaluate the health impacts of refined and whole grains, we need to acknowledge that each of them has some benefits and potential harms. Refined grains can increase glycemic load, heightening the risk of insulin resistance [40,41,42], but they are easier to digest than whole grains. Whole grains provide more nutrients than refined grains but contain gluten and lectin which can increase intestinal inflammation and barrier permeability [43,44]. Small amounts of whole grains consumed along with refined grains can help reduce glycemic load, but large amounts of whole grains add extra burden to those with impaired gut function. Our study is the first to demonstrate that large amounts of whole grains worsen the adverse effects of refined grains (1.52; 95% CI, 1.07 to 2.14). Whole grains include many types, such as wheat and oats, and different nutrients such as gluten. From clinical point of view, people who are wheat or gluten sensitive may not have celiac disease but do present clinical symptoms [45]. Whether cancer treatment can increase the prevalence of wheat or gluten sensitivity in breast cancer survivors has not been well studied. Moreover, the damages to digestive system caused by cancer treatment may or may not be the same as people who are wheat or gluten sensitive. Our study emphasizes the need to evaluate the adverse impacts of refined grains together with whole grain consumption. More detailed assessments of the clinical symptoms after whole grain consumption in breast cancer survivors are needed.

This study has several strengths. Our findings are the first to investigate the individual and joint associations of whole grains and refined grains with mortality among breast cancer survivors. We collected 24 h recalls four times during each visit, which is a unique strength of this study as it can reduce within-person variability and measurement errors. Most cohorts either do not have or have only one or two 24 h recalls. The large sample size provided us ample power to adjust for multiple confounding factors. Furthermore, the follow-up period of our study exceeds that of many prior studies, where grain consumption and mortality were investigated between 2 to 16 weeks [37]. 

There are several limitations of our study. Given that our study focused solely on breast cancer survivors, the generalizability of its results to survivors of other types of cancers will need to be confirmed by future studies. Since the women in our study were primarily white (80%) and enrolled in the study within 4 years of diagnosis rather than immediately after treatment, our study should not be generalized to other ethnic groups, or to those recently diagnosed with cancer. The 6-year follow-up period of the WHEL study restricted our ability to examine the extended associations of grain consumption on mortality beyond 6 years. More longitudinal studies are required to establish the causal associations between the type/quantity of grain consumed and mortality among breast cancer survivors. Furthermore, the 24 h recalls for each participant were self-reported, leaving room for errors in reporting of dietary information and the potential for recall bias. Finally, the digestive burdens caused by whole grain consumption also depend on the cooking methods; however, this was not recorded in the WHEL cohort as well as other cohorts. More rigorous designs are needed for studying whole grain consumption.

## 5. Conclusions

Our study bridged a critical gap in our understanding of the nutritional needs of female breast cancer survivors in the US as we are the first to investigate the independent and joint effects of whole/refined grain on total mortality among this population. Our findings demonstrate a significant and linear association of refined grain intake with total and breast-cancer-specific mortality, an exacerbation of the adverse effects with higher whole grain consumption, and the need to account for gastrointestinal health conditions of breast cancer survivors to formulate dietary guidelines for them. More robust observational studies should be conducted to replicate our study’s findings and to evaluate whether the adverse impacts of whole grain consumption vary across different ethnic groups or whether the consumption of whole and refined grain together with other food groups alters their effect on the overall health of breast cancer patients. 

## Figures and Tables

**Table 1:** Baseline demographics of the WHEL study participants (*n* = 3081).

	Overall	Mortality Status		*p*-Value
		No (*n* = 2767)	Yes (*n* = 314)	
Age at diagnosis (years)	50.7 ± 8.85	50.66 ± 8.7	51.45 ± 10.08	0.13
Age at randomization (years)	52.7 ± 8.97	52.63 ± 8.81	53.20 ± 10.29	0.30
BMI (kg/m^2^)	27.25 ± 6.07	27.16 ± 6.01	28.06 ± 6.52	**0.01**
Physical activity (Mets)	839.04 ± 878.83	857.30 ± 891.21	678.16 ± 743.15	**0.0007**
Total calorie intake (kcal)	1717.64 ± 407.59	1719.39 ± 403.81	1702.22 ± 439.87	0.48
Randomization group (N, %)				0.59
Intervention	1534 (49.79)	1379 (49.84)	155 (49.36)	
Comparison	1547 (50.21)	1388 (50.16)	159 (50.64)	
Chemotherapy (N, %)				**0.02**
Yes	2154 (69.91)	1909 (68.99)	245 (78.03)	
No	925 (30.02)	856 (30.94)	69 (21.97)	
Radiation therapy (N, %)				0.72
Yes	1894 (61.47)	1701 (61.47)	193 (61.46)	
No	1183 (38.40)	1062 (38.38)	121 (38.54)	
Hormone status (N, %)				**0.0005**
ER+/PR+	1905 (61.83)	1742 (62.96)	163 (51.91)	
ER+/PR-	493 (16.00)	432 (15.61)	61 (19.43)	
ER-/PR+	618 (20.06)	532 (19.23)	86 (27.39)	
ER-/PR-	65 (2.11)	61 (2.20)	4 (1.27)	
Ethnicity (N, %)				0.32
White	2627 (85.3)	2368 (85.58)	259 (82.48)	
Non-white	454 (14.7)	399 (14.42)	55 (17.52)	
Education (N, %)				**0.0197**
High school or less	377 (12.24)	329 (11.89)	48 (15.29)	
Post high school to college	1912 (62.06)	1715 (61.98)	197 (62.74)	
Post college	792 (25.71)	723 (26.13)	69 (21.97)	
Employment status (N, %)				0.20
Employed	2210 (71.73)	2003 (72.39)	105 (33.44)	
Unemployed	853 (27.69)	748 (27.03)	207 (65.92)	
Marital status (N, %)				**0.0417**
Single	338 (10.97)	299 (10.81)	39 (12.42)	
Married	2154 (69.91)	1945 (70.29)	209 (66.56)	
Other	589 (19.12)	523 (18.90)	66 (21.02)	

Continuous variables are presented as mean ± SD; categorical variables are presented as N, % (column %). Abbreviations: ER = estrogen receptor; PR = progesterone receptor; BMI = body mass index; METs = metabolic equivalents; Kcal = kilocalorie. Bolded *p*-Values indicate significance using a significance level (α) of 0.05.

**Table 2:** Baseline characteristics of breast cancer survivors by quartiles of the baseline whole grain consumption.

	Whole Grains (Servings/Day)				
	Quartile 1	Quartile 2	Quartile 3	Quartile 4	*p*-Value
Refined Grains (servings/day)	2.13 ± 1.08	2.05 ± 1.04	1.80 ± 1.19	1.7 ± 1.02	**<0.0001**
Age at diagnosis (years)	50 ± 8.59	50 ± 8.87	51 ± 9.05	50 ± 8.89	0.31
BMI	26.62 ± 6.45	25.95 ± 6.06	26.07 ± 5.69	24.97 ± 6.01	**0.0002**
Physical activity (METs)	450 ± 876.08	555 ± 880.49	600 ± 803.98	750 ± 940.95	**<0.0001**
Total calorie intake (kcal)	1597 ± 424.69	1631 ± 380.67	1687 ± 382.54	1816 ± 403.72	**<0.0001**
Smoking status (N, %)					**0.0013**
Never	421 (25.66)	409 (24.92)	427 (26.02)	384 (23.40)	
Former	329 (25.84)	299 (23.49)	315 (24.74)	330 (25.92)	
Current	52 (37.96)	41 (29.93)	27 (19.71)	17 (12.41)	
Chemotherapy (N, %)					0.14
No	223 (24.11)	233 (25.19)	236 (25.51)	233(25.19)	
Yes	586 (27.21)	524 (24.33)	544 (25.26)	500 (23.21)	
Cancer stage (N, %)					0.46
Stage I	309 (26.01)	299 (25.17)	294 (24.75)	286 (24.07)	
Stage II	467 (26.85)	423 (24.32)	449 (25.82)	400 (23.00)	
Stage III	33 (21.43)	37 (24.03)	37 (24.03)	47 (30.52)	
Hormone status (N, %)					0.78
ER+/PR+	497 (26.09)	472 (24.78)	472 (24.78)	464 (24.36)	
ER+/PR−	132 (26.77)	111 (22.52)	125 (25.35)	125 (25.35)	
ER−/PR+	163 (26.38)	158 (25.57)	168 (27.18)	129 (20.87)	
ER−/PR−	17 (26.15)	18 (27.69)	15 (23.08)	15 (23.08)	

Continuous variables are presented as mean ± SD; categorical variables are presented as N, % (column %). Abbreviations: ER = estrogen receptor; PR = progesterone receptor; BMI = body mass index; METs = metabolic equivalents; Kcal = kilocalorie. Bolded numbers represent significant *p*-values at a significance level (α) of 0.05.

**Table 3:** Grain consumption in relation to total mortality and breast-cancer-specific mortality.

		Total Mortality		Breast-Cancer-Specific Mortality	
		Event	HR (95% CI)	Event	HR (95% CI)
Whole Grains (servings/day)	Range				
Quartile 1	≤0.5	142	Ref	102	Ref
Quartile 2	0.6–0.98	140	1.06 (0.74, 1.58)	110	0.99 (0.93, 1.06)
Quartile 3	0.99–1.56	146	1.30 (0.91, 1.86)	119	1.03 (0.96, 1.10)
Quartile 4	≥1.57	130	1.36 (0.94, 1.97)	105	1.02 (0.95, 1.09)
***P* for trend**			0.07		0.55
Refined Grains (servings/day)	Range				
Quartile 1	≤0.91	98	Ref	70	Ref
Quartile 2	0.92–1.53	141	1.18 (0.81, 1.71)	113	1.07 (1.00, 1.15)
Quartile 3	1.54–2.31	142	1.40 (0.95, 2.06)	107	1.16 (1.08, 1.25)
Quartile 4	≥2.32	177	1.74 (1.17, 2.59)	146	1.16 (1.08, 1.26)
***P* for trend**			**0.0047**		**<0.0001**

The above hazard ratios were adjusted for age at diagnosis, smoking status, menopause status, physical activity, body mass index, calorie and protein intakes and cancer characteristics, namely chemotherapy, radiation therapy, cancer stage, and hormone status. Whole grains and refined grains were mutually adjusted in the multivariable models. Bolded *p* for trend values indicate significance using a significant level of 0.05.
